# A Case of Anterior Spinal Artery Syndrome Caused by *Streptococcus mitis* Endocarditis

**DOI:** 10.1155/2018/9658120

**Published:** 2018-01-29

**Authors:** Aria Mahtabfar, Hamoon Eshraghi, Melroy D'Souza, William Berrigan, Kathleen Casey

**Affiliations:** ^1^Rutgers Robert Wood Johnson Medical School, New Brunswick, NJ, USA; ^2^Department of Medicine, Jersey Shore University Medical Center, Neptune, NJ, USA; ^3^Department of Infectious Disease, Jersey Shore University Medical Center, Neptune, NJ, USA

## Abstract

**Background:**

Infectious endocarditis (IE) typically occurs in the setting of intravenous drug use, prosthetic heart valves, or rheumatic heart disease. However, there are a few reports of IE occurring in the setting of immunosuppression secondary to cancer and/or chemotherapy. Here, we present a case of a cancer patient who developed anterior spinal artery (ASA) syndrome secondary to a septic embolus from IE.

**Case Presentation:**

A 78-year-old male with a history of gastroesophageal cancer treated with chemotherapy and radiation presented to the hospital after a fall at home. He reported experiencing dyspnea and orthopnea for two weeks prior to presentation. In the ED, his vital signs were stable, and his examination was significant for a flaccid paralysis of the right lower extremity. Diagnosis of septic emboli secondary to IE was made after the echocardiogram showed the presence of vegetations on the aortic valve, blood cultures were positive for *Streptococcus mitis*, and thoracic spine MRI was indicative of an infarction at T10.

**Discussion:**

This case highlights the presence of IE in the setting of cancer and chemotherapy. Although cancer is a rare cause of IE, clinicians must maintain a high index of suspicion in order to minimize the sequelae of IE.

## 1. Introduction

Infectious endocarditis (IE) characteristically occurs after endothelial damage to native heart valves and fibrin deposition, which is followed by thrombus formation that serves as a nidus for bacterial invasion [1]. In developing nations, IE is usually caused from rheumatic heart disease, while in developed nations IE is secondary to congenital heart malformations, degenerative valvular disease, intravenous drug use (IVDU), and cancer [1]. The organisms that cause IE are typically (80–90%) gram-positive bacteria, including *Staphylococcus*, *Streptococcus*, and *Enterococcus* species [1].

*Streptococcus mitis* (*S. mitis*), along with other viridans group streptococci (VGS), is a commensal bacterium in the oropharynx. It typically causes an asymptomatic and transient bacteremia in the immunocompetent host but can lead to IE in the setting of valvular disease [1]. In an immunocompromised host, *S. mitis* can lead to a clinically significant bacteremia and IE [2]. For instance, patients with malignancy and neutropenia are susceptible to severe bacteremia and VGS shock syndrome secondary to *S. mitis* infection [3, 4]. Interestingly, amongst over 600 neutropenic patients across both studies, there were no patients that developed clinically significant IE from *S. mitis* infection, indicating that IE may not occur in the setting of neutropenia.

Here, we present the case of a patient with a history of cancer and a normal neutrophil count who developed IE secondary to *S. mitis* infection leading to anterior spinal artery (ASA) syndrome.

## 2. Case Presentation

A 78-year-old male with a history of gastroesophageal (G-E) junction carcinoma, treated at the time with chemotherapy (capecitabine) and radiation, arrived in the emergency room after a fall at home. He got out of bed and began walking, and his right leg “gave out.” The patient denied any recent trauma, pain, or loss of consciousness. He reported having paroxysmal nocturnal dyspnea and orthopnea for the past few weeks and he also reported no history of valvular disease and intravenous drug use.

On presentation, his temperature was 98F, blood pressure was 118/53 mmHg, heart rate was 86 beats per minute, respiratory rate was 12 breaths per minute, and oxygen saturation was 94%. There were no murmurs, lungs were clear to auscultation, and he had a flaccid paralysis of the right lower extremity at and below the level of the hip. Sensation to pinprick, light touch, vibration, and proprioception were intact bilaterally. Patellar reflexes were 2^+^ and symmetric. Plantar reflex was upgoing on the right lower extremity.

**Figure 1:**
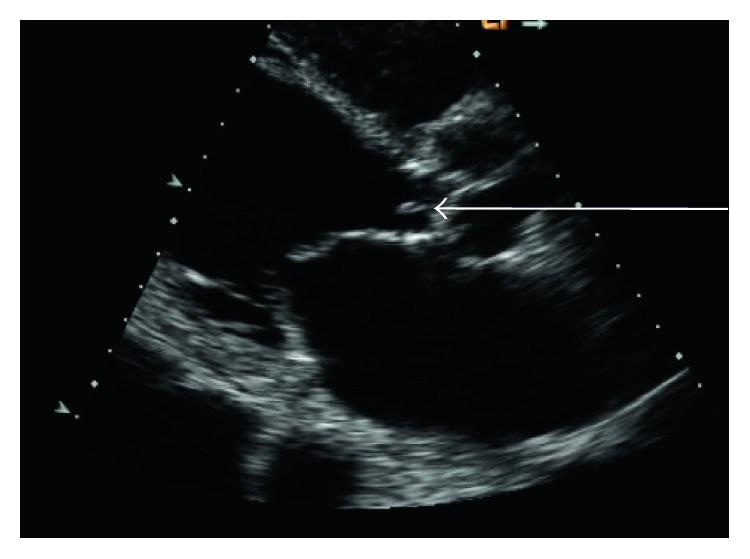
Transthoracic echocardiogram showing vegetation at the valley of the aortic valve cusp.

**Figure 2:**
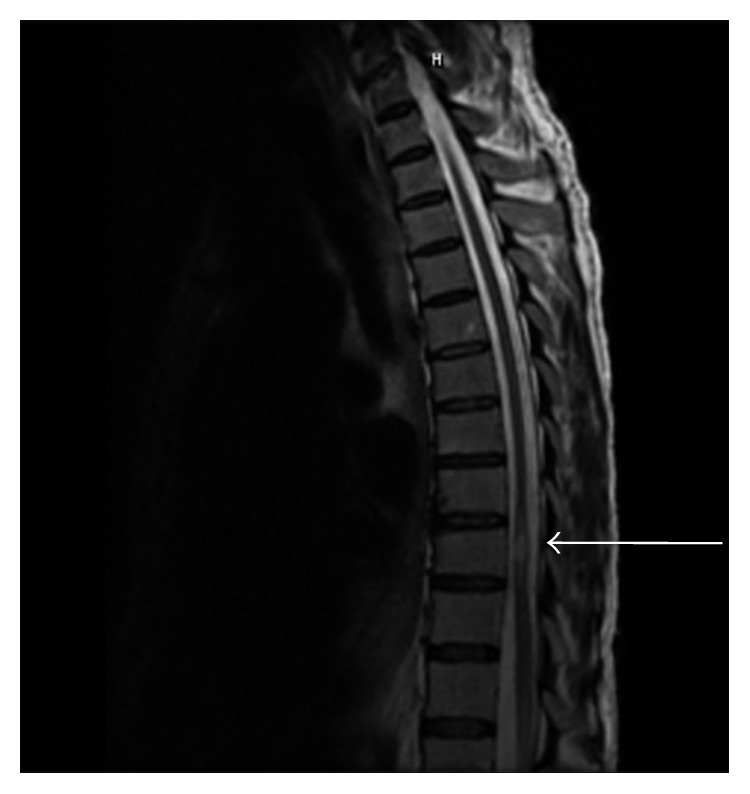
Focal area of abnormal signaling with nonenhancement at the level of T10.

Notable lab values were a brain natriuretic peptide > 5000 pg/mL (<100) and a white blood count of 13.8 × 10^3^/uL. A transthoracic echocardiogram revealed a mobile mass on the aortic valve, which was suspicious for vegetation (Figure 1). Blood cultures revealed the presence of gram-positive cocci in pairs and chains and were later shown to be *S. mitis*. Organism was identified via MALDI in addition to examining colony morphology and optochin sensitivity to help differentiate from other bacterial species. Brain magnetic resonance imaging (MRI) showed old ischemic changes. A thoracic spine MRI subsequently showed abnormal signaling at the level of T10 consistent with cord infarction (Figure 2).

The clinical picture was indicative of infective endocarditis, given that both the major criteria of the modified Duke criteria were met. Furthermore, the acute onset of focal neurological deficits was secondary to a septic embolus causing a spinal artery infarction. Vancomycin was initiated empirically and was changed to ceftriaxone based on the organism's antimicrobial sensitivities. After eight days of antibiotic therapy, blood cultures showed no growth of the organism. However, the patient's neurological condition worsened to a bilateral lower extremity paralysis and decreased sensation to touch, pain, and temperature bilaterally; vibration and proprioception sensation remained intact. He was discharged to a rehabilitation facility after two weeks and treated for an additional two weeks. Upon discharge, there was no improvement of his motor and sensory symptoms.

## 3. Discussion

We present a case of IE of a native aortic valve that subsequently dislodged a septic embolus and caused an occlusion of the artery of Adamkiewicz, leading to ASA syndrome. Given that this patient reported no history of valvular disease, rheumatic fever, or intravenous drug use, the emergence of IE was likely secondary to the immunosuppression caused by the G-E junction carcinoma and the subsequent chemotherapy. Although not a common cause of IE, cancer is a risk factor for IE in developed nations [1]. The association between *S. bovis* endocarditis and the development of colorectal cancer has been established for over 50 years; however, there remains a dearth of literature regarding the onset of endocarditis after the diagnosis and treatment of cancer [5]. The increased incidence of IE in cancer patients is in large part due to invasive techniques and indwelling devices, which was illustrated in one study which discovered that central venous catheters were responsible for almost a quarter of IE in cancer patients [6].

*S. mitis* is most commonly implicated in subacute endocarditis but can cause fulminant IE in the setting of immunosuppression as this patient likely had an attenuated immune response after receiving chemotherapy [7, 8]. As mentioned above, IE in cancer patients is commonly due to the use of catheters; however, the most common site of VGS in all patients is the gastrointestinal tract [9]. Therefore, it is also possible that the bacteria entered the patient's bloodstream after routine oral care or secondary to the G-E junction carcinoma and subsequent radiation therapy. Cancer and radiation cause a disruption in the integrity of the gastrointestinal tract's normal mucosal barrier, thereby allowing for the invasion of bacteria that would not normally penetrate into the blood. Once in the bloodstream, *S. mitis* can become clinically aggressive, lead to IE, and cause the release of several septic emboli in the immunocompromised patient [8].

In this patient, septic emboli from the aortic valve caused an infarction of the artery of Adamkiewicz, which is the largest spinal radicular artery and the only radicular artery to the ASA between T9 and T12 in most patients. While other regions of the spinal cord contain many collateral circulations with the ASA, this unique vascular supply makes the low thoracic region especially vulnerable to ASA syndrome [10]. The ASA supplies the anterior two-thirds of the spinal cord. Symptoms of ASA syndrome include complete motor loss below the level of the lesion as well as loss of pain and temperature sensation due to loss of the corticospinal and spinothalamic tracts, respectively; this patient exhibited findings consistent with ASA syndrome. Proprioception and vibratory sensation remained intact due to preservation of the dorsal columns, which are supplied by a pair of posterior spinal arteries. Septic emboli do not typically cause ASA syndrome, and the only documented case has been in an IVDU [11].

This case is the first presentation of ASA syndrome emerging secondary to *S. mitis* IE in a patient with malignancy [7]. Patients undergoing chemotherapy should be thoroughly assessed when there is any clinical suspicion for bacteremia or endocarditis given the likelihood of *S. mitis* infection, which may be resistant to penicillins and fluoroquinolones [7]. A recent study of pathogens in human breast milk showed that 83.3% of *S. mitis* isolates were resistant to at least one antibiotic, and of those, half were resistant to three or more antibiotics [12]. Furthermore, *S. mitis* can cause severe bacteremia in cancer patients with neutropenia. However, this case highlights that patients who have a normal neutrophil count should be monitored for IE as well. Therefore, we advise clinicians to maintain a high index of suspicion of *S. mitis* infective endocarditis given the high morbidity and mortality associated with the infection.

We look forward to future clinical vignettes regarding the development of IE in patients who are immunosuppressed from cancer and/or chemotherapy.
